# Accessing the Impact Mechanism of Sense of Virtual Community on User Engagement

**DOI:** 10.3389/fpsyg.2022.907606

**Published:** 2022-06-15

**Authors:** Hong Zhao, Qiaohong Shi

**Affiliations:** ^1^International College of Cultural Education, Northeast Agricultural University, Harbin, China; ^2^College of Finance and Economics, Nanchang Institute of Technology, Nanchang, China

**Keywords:** educational virtual community, sense of virtual community, affective commitment, perceived support, user engagement

## Abstract

Although research has begun to explore the influence patterns of sense of virtual community, there is limited research on how sense of virtual community affects educational virtual community user engagement. Based on the educational virtual community context, this study constructs a theoretical model with moderation and mediation to explore the mediation mechanism of sense of virtual community affecting user engagement and its boundary conditions. In this study, the data collected from 377 users are analyzed by structural equation modeling. The research findings found that not only effective commitment has a mediating role between sense of virtual community and user engagement, but also perceived support has a moderating role in the process of effective commitment’s influence on user engagement. This study examines the practical effects of sense of virtual community in the context of educational virtual community use and reveals the mechanism of the effect of sense of virtual community on user engagement.

## Introduction

The development of Coronavirus disease 2019 (COVID-19) has changed the way the public lives, communicates and learns. In the context of COVID-19, the application of new information technology has dramatically changed the way people learn and interact with each other, and the educational virtual community has flourished and set off a profound change in the education industry. Cloud classroom, Massive Open Online Courses (MOOC) and other educational service products based on digital content and technology have emerged one after another. In educational virtual communities, sense of virtual community is the key to effective online learning and an important antecedent variable influencing the integration of members into the educational virtual community (Liu et al., 2020). In recent years, empirical studies around the sense of virtual community has been highly sought after by marketing researchers (Koh and Kim, 2003; Brodie et al., 2013; Chou et al., 2016; Zhang et al., 2021). Related studies have focused on consumers’ subjective perceptions of specific virtual communities, emphasizing the extent to which consumers’ belonging, identity, and attachment to their virtual communities, and their behavioral performance in establishing and creating a positive community atmosphere and mimicking real situations, all play an important role in influencing and enhancing their subsequent attitudes and behaviors in virtual environments (Tsai et al., 2011; Petric, 2014). The results of the current study show that the sense of virtual community affects user engagement in educational virtual community, including word-of-mouth promotion, effective commitment, creative feedback, conveying emotion, developing awareness, and conducting interaction (Chou et al., 2016; Lu and Hu, 2017; Hu et al., 2019; Liu et al., 2020).

Such a number of effects lead to a strong or intense user engagement when the educational virtual community revaluates the sense of virtual community (Liu et al., 2020). In other words, sense of virtual community can have a very significant positive impact on user engagement. Sense of virtual community is the essence of establishing and developing communities in virtual environments (Han et al., 2016), reflecting the behavioral manifestations outside the value purchase of virtual community consumers. Sense of virtual community is to create a relational link between consumers and the educational virtual community. Research has found that sense of virtual community can motivate community members to actively participate in community activities and create brand value with the educational virtual community (Chou et al., 2016). However, fewer studies have explored and examined the mechanisms and boundary conditions underlying the effect of sense of virtual community on individual-level behavioral performance. Therefore, given the important role of user engagement on the construction of educational virtual community (Tarute et al., 2017; Ul Islam et al., 2019), this study builds on existing relevant studies from the educational virtual community perspective to examine the potential intermediate mechanisms by which sense of virtual community affects user engagement and provide relevant empirical research support.

More importantly, as a psychological state reflecting the relationship between members and the educational virtual community, affective commitment reflects the willingness of members to be part of the organization on an ongoing basis (Allen and Meyer, 1990). In educational virtual communities, in the absence of rules and regulations governing members’ activities, commitment mechanisms become an important alternative system for managing community relationships and members’ behaviors (Zhou et al., 2012). A direct relationship between effective commitment and user engagement has been shown (Raies et al., 2015), but the formation of effective commitment relies on the positive climate and embedded resources that the educational virtual community fosters for consumers. atmosphere and embedded resources provided by the educational virtual community (Mamonov et al., 2016).

The research also shows that effective commitment, as a psychological state that characterizes the relationship between members and the educational virtual community, reflects the long-term orientation of the relationship, guiding and regulating consumer behavior and making consumers act more based on the common interests of both parties (Zhu and Xie, 2018; Pang and Yang, 2021; Yang et al., 2021). Therefore, effective commitment is necessarily a prerequisite for user engagement educational virtual community and may play a mediating role in the relationship of sense of virtual community to user engagement. Currently, there are few studies linking sense of virtual community, effective commitment, and user engagement in studies related to consumer behavior, especially the lack of attention to the mediating role of effective commitment. Therefore, this paper proposes that the sense of virtual community created by the educational virtual community may have an indirect positive effect on the user engagement of the educational virtual community through the mediating role of effective commitment indirect positive effect on user engagement.

In addition, the stronger user experience means that individuals perceive that the educational virtual community values Individuals’ contributions to the educational virtual community and the level of concern for user welfare, which not only provides a support behavior for consumers in valuing content quality, encouraging interactions between individuals, but also to promote the participation of members in the various incentives set by the educational virtual community (Marique et al., 2013). Previous research on user perceived support has found that when companies work hard for a good user experience, in return, individuals contribute to the betterment of the company (Bettencourt, 1997; Ashley et al., 2010). Rosenbaum (2006) argues that when a company’s efforts to satisfy its Individuals are recognized by them, then the company must be special in the minds of its Individuals and the company can get certain economic benefits from user satisfaction. Rosenbaum and Massiah (2007) found in a study of user buying behavior that user perceived support is effective in reducing user perceived risk, enhancing user recognition of the company, and thus promoting buying behavior. The various user support tools provided by companies help Individuals have positive psychological experiences and enhance their relationship commitment to the company (Zhao et al., 2014), which has a significant positive impact on user trust and thus increases Individuals’ willingness to share information (Wang and Rao, 2015). Existing research results (Ning and Xue, 2016; Meng, 2017; Wang et al., 2019) show that perceived support is effective in enhancing user attitudes and behaviors, which in turn brings visible benefits to companies. However, in the existing research on perceived support, scholars have mostly explored the issue from the perspective of employees and organizations, while few studies have been conducted on consumers’ perceived support of live streaming platforms in the specific context of education. Therefore, this study introduces the issue of user perceived support into the field of virtual community marketing for exploration, which has certain theoretical significance to enrich the theory of virtual community marketing. Therefore, this study introduces perceived support as a moderating variable, analyzes the mechanism of perceived support in educational virtual community, and provides recommendations for educational virtual community managers to strengthen the construction of educational virtual community through perceived support.

## Theoretical Foundation

### Educational Virtual Community

Rheingold (1993) first proposed the use of virtual communities to describe social groups with common values and interests on the Internet, and today virtual communities are widely used in education, and virtual educational communities have become an important part of information-based teaching and learning. In virtual education communities, educators and learners can communicate and discuss pedagogical aspects through information exchange platforms, instant messaging software and other network communication services for the purpose of knowledge creation and knowledge sharing. Virtual education communities are a useful supplement to traditional education methods, which not only break the limits of classroom teaching, extend learning time, and help learners, develop independent learning skills, but also provide a more flexible and personalized way of cognition. With the in-depth development of big data technology, the rise and popularity of iconic social networking tools such as Tik Tok and WeChat have led people to increasingly socialize and interact through social networks. Interactive learning based on these social networking tools not only successfully situates the collaborative and mutually beneficial processes of traditional learning communities in virtual social contexts and builds virtual educational communities, but also allows such virtual educational communities to incorporate the benefits of social networking (Junco et al., 2011). Social network-based virtual educational communities promote the natural occurrence of cognitive behaviors, but also have the rapidity and linguistic nature of interactive communication, which encourages interaction among community members in terms of knowledge collaboration, resource sharing, and experience exchange, effectively advancing learners’ cognitive behaviors in the learning process.

A core component of traditional communities is a sense of belonging to a group. In a community there is a sentiment that members are attached to the group and to each other and share a common belief that their needs can be met through the obligation of members to be together (Xu and Wang, 2007). Educational virtual communities, on the other hand, describe groups of learners who share common learning interests on the Internet. Because the construction of virtual educational communities is both related to network and software technologies (Sun and Gao, 2008) and inextricably linked to interpersonal interactions, a deeper understanding of virtual educational communities is advocated through both technical and social levels; from the technical level, virtual educational communities present learners’ psychological experiences that are mediated by communicative media devices, which is a technical phenomenon; from the social level, virtual educational communities also embody communicative interactions among groups of learners, which is a social phenomenon. Therefore, compared to traditional learning communities, elements of virtual educational communities include not only a sense of belonging to a group of learners and common goals, but also network technologies and interactive behaviors based on network technologies.

### Sense of Virtual Community

The sense of virtual community has long been a hot topic of academic and practical attention as a description of members’ subjective feelings toward a specific virtual community. Studies have shown that sense of virtual community can motivate community members to actively participate in community activities, promote collaboration among virtual community members, increase knowledge and information sharing among community members, convert non-online activities of community members into online activities, and increase loyalty of e-commerce consumers (Koh and Kim, 2003; Blanchard and Markus, 2004). First, the positive impact of sense of virtual community on social support, sense of virtual community is an important mechanism for people’s behavioral responses when faced with input or political mobilization pressure in online environments (Wang, 2010; Daffern et al., 2021). Obst and Stafurik (2010) found that in virtual communities, online community support and a sense of community existed among members, and that by participating in virtual community members could receive moral support and personal advice. In other words, a strong sense of community will predict higher levels of mobilization and intention to act if mobilization pressure from online groups is consistent with the beliefs and values that individuals possess (Tsai et al., 2012). Second, the sense of virtual community has greater explanatory power for trust. Sense of virtual community plays an active role in promoting mutual trust among community members, and trust among members is an important impact outcome of sense of virtual community (Blanchard et al., 2011). That is, members’ community feelings reinforce their beliefs about the trustworthiness of other community members; at the same time, the presence and adherence to norms in the virtual community leads to a stronger sense of virtual community and ultimately to a strong sense of trust in other members of the online community. In addition, Tsai et al. (2011) studied the effects of technology acceptance factors and social factors on online group purchasing and showed that sense of virtual community can lead to positive outcomes such as increased satisfaction, increased community communication, increased trust, and increased social interaction. In addition, the sense of virtual community has a facilitating effect on sustained engagement (Chai and Kim, 2012).

As a complex construct and a rather novel direction of research, many scholars have defined the multidimensional concept of sense of virtual community. Roberts et al. (2002) explored the sense of virtual community in chat rooms, and they found through their qualitative study that the virtual communities they examined, although different from face-to-face communities, members still experience a sense of community similar to that defined by McMillan and Chavis (1986). Among them, Koh and Kim’s (2003) view is the most representative and widely accepted. They argue that sense of virtual community is a sense of belonging to the community, a feeling between members and between members and the community, and a shared belief that members’ needs will be met. Sense of virtual community in the context of educational virtual community is expressed in the dimensions of membership, influence, and immersion (Koh and Kim, 2003). Specifically, membership reflects community members’ sense of belonging to the virtual community; influence refers to community members’ perception of influencing and being influenced by others; and immersion refers to the amount of time and energy community members spend in the community beyond the usual level, describing a degree of community involvement. Based on existing studies (Tsai et al., 2011, 2012; Chen et al., 2013; Han et al., 2016), this study argues that sense of virtual community is essential to the establishment and development of virtual communities and not only shows a significant contribution to affective bonding, but also can be considered as a key antecedent to integration into virtual communities. The sense of virtual community given to members by the educational virtual community can drive the achievement of this high level of emotional bonding.

## Research Model and Hypotheses

### Sense of Virtual Community and Affective Commitment

It has been shown that membership is often used to explain the willingness and commitment of users of virtual communities to maintain long-term relationships with virtual communities (Dholakia et al., 2004). Previous studies have found that membership is an important antecedent factor that influences active participation of community members. For example, Hsu and Lin (2008) found that membership motivated members to be more active in writing blog content, giving feedback on their product suggestions, and actively promoting the product. In educational virtual communities, membership is related to the degree of affective commitment of members. Hashim and Tan (2015) argue that focusing on affective commitment, the empirical results based on online business communities show that affective commitment makes consumers see themselves as “hosts” of the community and thus more willing to share knowledge and spread brand-related information. Membership is an important factor in maintaining the relationship between members and the educational virtual community, which helps to stimulate members’ interest in the community. Membership is an important factor in maintaining the relationship between members and the educational virtual community, and is conducive to a high level of affective commitment to the community (Zhou et al., 2012). Obviously, it can be judged from this that the membership of the online educational virtual community has an influential role on the user affective commitment.

Research suggests that influence is seen as an important antecedent to affective commitment. Influence is often present in the specific educational virtual community in which members live and is seen by members as a specific partnering resource that can help them achieve their community goals. The emotional relationships and affective attitudes among members of the educational virtual community allow the relationship between members and the educational virtual community to develop from independent individual relationships to group relationships, and to generate attachment and identification with the educational virtual community. In the educational virtual community, influence is a mechanism that sustains the long-lasting relationship between the educational virtual community and its members, and the high prestige that members receive through their contribution is a spiritual reward that keeps their high level of affective commitment to the educational virtual community. The greater the influence of members in an educational virtual community, the more likely they are to become attached (Dennis et al., 2016). In other words, the influences of the members of the educational virtual community are positively related to their affective commitment to the educational virtual community. Furthermore, in an educational virtual community, influence is an embedded human resource that exists within the member’s relationship with that community and is difficult to be replicated to other educational virtual communities and is completely lost when the member leaves the community (Tiwana and Bush, 2005). Therefore, the greater the member influence, the greater the tendency to generate high levels of affective commitment to the educational virtual community. Clearly, it can be judged that the online educational virtual community influence has an impact on user affective commitment has an influential role.

It was found that immersion implies that members invest time and energy in the virtual community beyond the norm. On the one hand, the high level of time and energy invested by educational virtual community members leads to positive attitudes toward the community (Zhou et al., 2012). In other words, immersion leads to a high level of affective commitment to educational virtual community. Dai and Salam (2014) argue that the interaction between members in the virtual environment, as well as between members and service providers, is very frequent, and the interaction that members The immersive experience that members feel during this interaction is even more influential in their choice of service provider and the level of affective commitment. On the other hand, the time and effort that members invest in the educational virtual community is a sunk cost, and only by paying this cost is it possible to build relationships with other members in the educational virtual community. In order to avoid the cost loss, members will have a high level of affective commitment to the educational virtual community (Dai and Salam, 2014). In other words, members of the educational virtual community maintain their relationship with the educational virtual community through affective commitment out of consideration for the benefits they pay. Obviously, it can be judged from this that the immersion of the online educational virtual community has an influential role on users’ affective commitment. Based on the aforementioned arguments, this study hypothesizes the following:

**Hypothesis 1:** There is a positive relationship between sense of virtual community and affective commitment.

**Hypothesis 1a:** There is a positive relationship between membership and affective commitment.

**Hypothesis 1b:** There is a positive relationship between influence and affective commitment.

**Hypothesis 1c:** There is a positive relationship between immersion and affective commitment.

### Affective Commitment and User Engagement

Affective commitment is a key component of commitment and is an important topic in the field of social exchange and marketing as an important factor influencing long-term relationships (Lee et al., 2018; Sun, 2021). In their study of organizational commitment, Meyer and Allen (1991) considered affective commitment as the tendency of employees to identify with, commit to, and emotionally attach to the organization and thus psychological tendency to become supportive of the organization. It has been shown that members engage in community activities because they feel a strong emotional connection to the community (Chang and Chuang, 2011), and that this emotional experience also generates strong user engagement (Ahn and Back, 2018). In an educational virtual community, affective commitment is a physical emotion, a positive psychological disposition formed by members being involved in a virtual community and participating in the activities of that virtual community (Schulten and Schaefer, 2015). Affective commitment reflects a membership mind-set that emphasizes a willingness to contribute to the achievement of community goals (Bateman et al., 2011). When members of an educational virtual community interact with an educational virtual community, they generate positive emotions toward that educational virtual community, and this emotion induces the user’s affective commitment, which in turn influences members to interact and integrate again with the educational virtual community (Casaló et al., 2008; Jayasingh, 2019), and user engagement is the best expression of interaction between members and the educational virtual community is the best expression of interaction between members and the educational virtual community. Therefore, when members develop a high level of affective commitment to the educational virtual community, they are induced to develop a high level of inclusion behavior. In other words, the emotional connection and relational commitment between consumers and the educational virtual community positively contribute to the formation of user engagement. Apparently, effective commitment determines the level of integration achieved by members. Brodie et al.’s (2013) study pointed out that harmonious and friendly relationships are potential antecedents of integration and important value outcomes of integration and prerequisites for achieving higher levels of integration behaviors, and effective commitment is a reflection of harmonious and friendly relationships between members and the effective commitment is a reflection of the harmonious and friendly relationship between members and the educational virtual community. Obviously, it can be reasoned that affective commitment has a positive influence on user engagement educational virtual community. As such, this study expects that brand identity will drive consumers to attain higher purchase intention.

**Hypothesis 2:** There is a positive relationship between affective commitment and user engagement.

### Mediating Role of Affective Commitment

Sense of virtual community can influence affective commitment in a general sense and promote user engagement, so effective commitment plays a mediating role in the relationship between sense of virtual community and user engagement. In educational virtual communities, affective commitment reflects the behavioral intention of members to establish and maintain long-term relationships with the virtual community (Chang and Chuang, 2011), implying that consumers are attached to the educational virtual community (Yang et al., 2014). When members form a high level of affective commitment to the educational virtual community, they not only maintain and support the activities organized by the educational virtual community (Casaló et al., 2008), but also make virtual community, they will also engage in integration behaviors such as advocacy (Shukla et al., 2016), advocacy (Sashi, 2012), and loyalty. High levels of affective commitment cannot be formed without the experience brought to consumers by the educational virtual community and consumers’ feelings about the formation of the virtual community (Jang et al., 2008; Shen et al., 2018). Clearly, affective commitment plays a mediating variable in the relationship between sense of virtual community and user engagement in educational virtual community.

First, when members develop membership in the educational virtual community, it induces a sense of obligation to the community, and consumers see themselves as part of the educational virtual community, and user engagement becomes an active behavior. Second, the greater the influence of the educational virtual community, the more members tend to occupy the central position of the educational virtual community, and the stronger the emotional connection to the educational virtual community, and the more likely they are to generate The greater the community influence, the more members tend to occupy the central position of the educational virtual community, the stronger the emotional connection to the educational virtual community, and the more likely to generate affective commitment and further generate high levels of user engagement. Third, when members participate in more community activities and immerse themselves in the educational virtual community, they will acquire richer knowledge and experience through these activities and become the object of consultation by other community members, and they will have stronger affective commitment to the educational virtual community as well as other members. This leads to positive user engagement behaviors, such as solving problems for other members and the educational virtual community.

Obviously, membership creates a high level of affirmation of the educational virtual community, and in order to maintain this good relationship, the members concerned will actively promote the development of the educational virtual community and collaborate in its operation in order to strengthen the integration of the educational virtual community (Cheng and Guo, 2015). Influence makes the time and energy spent by members on the educational virtual community an asset, and this asset creates a lock-in effect, i.e., members’ immersion in the educational virtual community makes it difficult for them to leave and continue to create value for the educational virtual community. Thus, in educational virtual communities, sense of virtual community has an impact on inclusion because sense of virtual community creates a connection. In other words, there is a mediating variable between sense of virtual community and user engagement, i.e., affective commitment. Comprehensive analysis of the above, this study thus hypothesizes:

**Hypothesis 3:** The affective commitment mediates the relationship between sense of virtual community and user engagement.

**Hypothesis 3a:** The affective commitment mediates the relationship between membership and user engagement.

**Hypothesis 3b:** The affective commitment mediates the relationship between influence and user engagement.

**Hypothesis 3c:** The affective commitment mediates the relationship between immersion and user engagement.

### Moderating Role of Perceived Support

Perceived support is the degree to which Individuals perceive that the company cares about them and values their contributions (Bettencourt, 1997). Organizational support theory suggests that a company must support and help its employees adequately if it wants them to contribute voluntarily (Chen and Liao, 2006; Yang et al., 2009). The higher the perception of organizational support employees feel, the better the relationship between the employee and the organization, and the more effective commitment will be significantly enhanced (Meyer and Smith, 2000). Social exchange theory states that individuals tend to take positive actions in return for positive treatment from the giver (Surma, 2016). Therefore, when users perceive that the educational virtual community is working hard to give them a perfect experience, they will also take positive actions, such as generating user engagement, for the educational virtual community to achieve better development and profitability. As a result, perceived support makes users feel positive about the educational virtual community, and the stronger the perceived support is, the more it stimulates users’ sense of ownership and responsibility, and the more it makes them willing to do “voluntary.” The stronger the perceived support is, the more it stimulates a sense of ownership and responsibility, and the more willing users are to engage in voluntary behavior. When users perceive that their interests are valued or cared for by the company, they will take the initiative to pay attention to the future development of the company and make contributions accordingly (Wu, 2011).

Research has been conducted to examine the impact of user-generated affective commitment on user behavior. Yin and Zhang (2020) showed that promoting affective commitment positively influences user citizenship behavior; compared to low levels of perceived support, high levels of perceived support enhances the positive impact of affective commitment on user behavior compared to low perceived support. The results of this study not only provide a theoretical basis for the relationship between affective commitment and user behavior, but also provide practical insights for companies and related managers, as well as evidence for the moderating role of perceived support. Based on this, when users perceive a higher sense of support from the educational virtual community, they feel that the educational virtual community values and recognizes them, which makes them feel emotionally satisfied and fond of the educational virtual community they are in, thus gaining a sense of belonging and dependence and prompting them to make consumption behaviors. In addition, the perceived support also triggers the users’ affective commitment, in which the users’ incorporation of the target object is enhanced, which in turn leads to consumption intention. Based on the above analysis, this study inferred that perceived support has a moderating role in the influence of affective commitment on user engagement.

**Hypothesis 4:** Perceived support plays a positive moderating role between affective commitment and user engagement.

**FIGURE 1:**
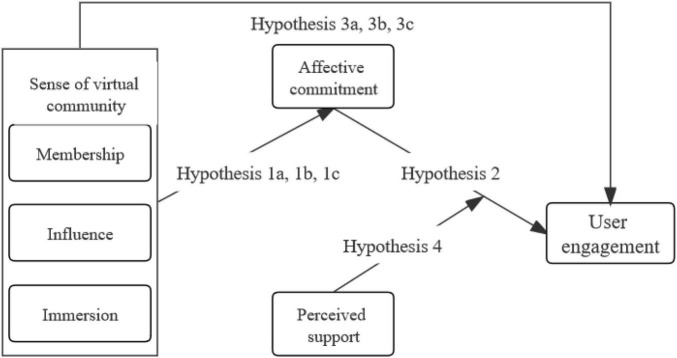
Theoretical model.

The research model for this study is shown in Figure 1.

## Materials and Methods

### Participants and Procedure

In this study, “LiZhi Microclass” is used as the subject of the survey, because “LiZhi Microclass” is a high-end education virtual platform and a search consumer product, and consumers usually consider many factors when making decisions, thus prompting them to learn about the integrated educational virtual community and its enterprises through various channels, and the information related to the sense of educational virtual community may enter the knowledge structure and cognitive scope of consumers. “LiZhi Microclass” is a live online training platform based on WeChat, which is the most popular online training platform in history. It is also an online education platform that attracts web celebrity lecturers to share their knowledge, supports 100,000 people online at the same time, reviews past content at any time, smoothly and without restrictions, and makes knowledge transfer more convenient. There are five major modes: ppt + voice interactive, graphic+voice interactive, video+voice interactive, video recording, audio recording. Obviously, “Lychee Microclass” is a typical representative of educational virtual community. In addition, as a domestic industry with relatively large growth potential, if the findings of this study can be supported by the data of “LiZhi Microclass,” it can in turn provide management suggestions for the marketing activities of the educational virtual community companies. In this study, online questionnaires were distributed to users through the “private chat” function of “LiZhi Microclass,” and online questionnaires were distributed to users of “LiZhi Microclass” through social channels such as WeChat and forums, with rewards in the form of RMB 5 for completing the questionnaires. The questionnaire survey of this study started from September 2021 and ended in February 2022, which lasted for 6 months, and 430 questionnaires were finally collected, of which 53 were invalid (i.e., questionnaires that selected “have not used LiZhi Microclass”, checked the same option for the main question, and had inconsistent answers to questions), and 377 were valid. The valid questionnaire rate was 87.67%. Table 1 shows the demographic characteristics of the valid sample.

**TABLE 1:** Descriptive statistical analysis.

Variables	Item	Frequency	%	Cumulative%
Gender	Male	186	49.3	49.0
	Female	191	50.7	100.0
Age (year)	19 or less	11	2.9	2.9
	20∼29	165	43.8	46.7
	30∼39	147	39.0	85.7
	40∼49	41	10.9	96.6
	50 or above	13	3.4	100.0
Marriage	Married	227	60.2	60.2
	Unmarried	147	39.0	99.2
	Divorce	3	0.8	100.0
Professions	Student	60	15.9	15.9
	Freelance	25	6.6	22.5
	Executive in private enterprise	115	30.5	53.1
	Civil servant	155	41.1	94.2
	Clerk in state owned enterprise	18	4.8	98.9
	Executive in private enterprise	4	1.1	100.0
Education	College and blow	53	14.1	14.1
	Undergraduate	288	76.4	90.5
	Master’s degree and above	36	9.5	100.0
Consumption (RMB)	Below 2,000	36	9.5	9.5
	2,000∼3,999	65	17.2	26.8
	4,000∼5,999	91	24.1	50.9
	6,000 or more	185	49.1	100.0
Continuous use time (year)	Less than 1	62	16.4	16.4
	1∼2	162	43.0	59.4
	Over 3	153	40.6	100.0

### Measures

To ensure the content validity of the variables and their measurement items, existing established scales should be used directly or adapted to measure each variable in the research model as much as possible. To this end, all of the constructs in the conceptual model of this study were drawn from previously developed measurement scales and were measured using a 7-point Likert scale, ranging from “strongly disagree/strongly agree” and “very unimportant/very important. The measurement items were all appropriately textualized from the original literature based on the context in which this study was conducted. See Table 2 for details of the measurement scales used in this paper.

**TABLE 2:** Variables and measurement item.

Variables	Items	References
Membership	MEM1. I feel as if I belong to the LiZhi Microclass.	Koh and Kim, 2003; Chou et al., 2016
	MEM2. I sense my membership in the LiZhi Microclass.	
	MEM3. I feel as if the LiZhi Microclass members are my close friends.	
	MEM4. I like the members in the LiZhi Microclass.	
Influence	INF1. I am well-known as a member of the LiZhi Microclass.	Koh and Kim, 2003; Chou et al., 2016
	INF2. I feel that I control other members in the LiZhi Microclass.	
	INF3. My postings in the LiZhi Microclass are often reviewed by other members.	
	INF4. Replies to my postings appear in the LiZhi Microclass frequently.	
Immersion	IMM1. I spend much time online in the LiZhi Microclass.	Koh and Kim, 2003; Chou et al., 2016
	IMM2. I spend more time than I expected navigating the LiZhi Microclass.	
	IMM3. I feel as if I am addicted to the LiZhi Microclass.	
	IMM4. I have missed classes or work because of the LiZhi Microclass activities.	
Affective commitment	AC1. When I use LiZhi Microclass, I immerse myself unconsciously.	Yang et al., 2021
	AC2. I have a deep affection for the LiZhi Microclass.	
	AC3. LiZhi Microclass gives me a strong sense of belonging.	
	AC4. The LiZhi Microclass is very attractive to me.	
Perceived support	PS1. The LiZhi Microclass strongly considers my needs and wants.	Wu, 2011
	PS2. Help is available from LiZhi Microclass when I have a problem.	
	PS3. The LiZhi Microclass tries to provide the best service possible.	
	PS4. The LiZhi Microclass is willing to help me when I have a special request.	
User engagement	CE1. Whenever I have to use educational virtual community, I usually use LiZhi Microclass.	Brodie et al., 2013; Hollebeek et al., 2014
	CE2. I am passionate about the LiZhi Microclass.	
	CE3. I love the LiZhi Microclass.	
	CE4. I am excited when using the LiZhi Microclass.	

### Data Analysis Methods

This paper examines the impact of sense of virtual community on user engagement in the context of educational virtual community, with the main objective of extending the existing sense of virtual community to new application areas. As such, it is a validation study, suitable for data analysis by means of structural equation modeling (SEM). More importantly, SEM has rigid restrictions on the number of samples and whether they obey multivariate normal distribution, which facilitates the analysis of structural relationships between multiple independent and dependent variables. Obviously, it is more appropriate to use Amos 24.0 statistical software to test the data in this paper.

## Data Analysis Results

### Measurement Model Analysis

This study evaluates and revises the Confirmatory Factor Analysis (CFA) measurement model based on a two-stage model (Kline, 2011). Currently, academics generally agree with the approach of Anderson and Gerbing (1988). That is, CFA should report Factor Loading, Cronbach’s Alpha, Composite Reliability (CR), and Average Variance Extracted (AVE) for all variables, and only after these metrics pass the test can structural models be evaluated. Specifically, Factor Loading > 0.50, Cronbach’s Alpha > 0.70, CR > 0.60, and AVE > 0.50 (Fornell and Larcker, 1981; Anderson and Gerbing, 1988; Nunnally and Bernstein, 1994; Hair et al., 2017), then the measurement model has good convergence validity. The results of the CFA are shown in Table 3. Among them, factor loadings of all dimensions are between 0.614 and 0.931, Cronbach’s Alpha is between 0.814 and 0.866, and CR is between 0.818 and 0.865. AVE is between 0.531 and 0.625, indicating that each construct has good convergence validity.

**TABLE 3:** Confirmatory factor analysis.

Variables	Items	Factor loadings	Cronbach’s alpha	CR	AVE
Membership	MEM1	0.677	0.866	0.865	0.618
	MEM2	0.784			
	MEM3	0.841			
	MEM4	0.832			
Influence	INF1	0.872	0.852	0.860	0.608
	INF2	0.741			
	INF3	0.807			
	INF4	0.686			
Immersion	IMM1	0.697	0.814	0.818	0.531
	IMM2	0.616			
	IMM3	0.790			
	IMM4	0.797			
Affective commitment	AC1	0.651	0.852	0.853	0.596
	AC2	0.716			
	AC3	0.843			
	AV4	0.858			
user engagement	UE1	0.614	0.858	0.865	0.625
	UE2	0.633			
	UE3	0.926			
	UE4	0.931			
Perceived support	PS1	0.812	0.854	0.854	0.596
	PS2	0.841			
	PS3	0.751			
	PS4	0.674			

Table 4 reports the Discriminant validity for the measurement model, the square roots of the AVE are reproduced on the diagonal. Discriminant validity is the extent to which the measure is not a reflection of some other variables. It is indicated by low correlations between the measure of interest and the measures of other constructs. This study has examined discriminant validity using Fornell and Larcker (1981)’s recommendation that the square root of the average variance extracted for each construct should be higher than the correlations between it and all other constructs. Table 4 shows that the squared root of average variance extracted for each construct is greater than the correlations between the constructs and all other constructs. The results support Fornell and Larcker (1981)’ requirement of discriminant validity.

**TABLE 4:** Discriminant validity for the measurement model.

Variables	Mean	SD	AVE	1	2	3	4	5	6
1. Membership	4.607	1.197	0.618	0.786					
2. Influence	3.889	1.210	0.608	0.437	0.780				
3. Immersion	4.221	1.073	0.531	0.523	0.538	0.729			
4. Affective commitment	4.932	1.126	0.596	0.533	0.526	0.585	0.772		
5. User engagement	5.529	0.871	0.625	0.307	0.226	0.261	0.446	0.791	
6. Perceived support	4.405	1.181	0.596	0.357	0.271	0.516	0.471	0.219	0.772

*The diagonal value is the square root of AVE.*

### Structural Model Analysis

In a previous study, they found in 194 papers of international academic journals, there are nine most commonly reported model fit indices (Jackson et al., 2009). As suggested by Jackson et al. (2009), MLχ2, DF, Normed Chi-sqr (χ2/DF), RMSEA, SRMR, TLI (NNFI), CFI, GFI, and AGFI are the common metrics used to test the fit of research models. In SEM analysis, if the sample size is larger than 200, it will cause chi-square to inflate leading to decreased model fit (Bollen and Stine, 1992). This study used Bollen-Stine Bootstrap to corrected SEM chi-square. After Bollen-Stine bootstrapping correction, the model fits indices fit all the criteria of suggestions as Table 5 shown.

**TABLE 5:** Model fit criteria and the test results.

Model fit	Criteria	Model fit of research model
χ^2^	The small the better	201.793
DF	The large the better	163
Normed Chi-square(χ^2^/DF)	1 < χ^2^/DF < 3	1.248
RMSEA	<0.08	0.025
SRMR	<0.08	0.023
TLI (NNFI)	>0.9	0.989
CFI	>0.9	0.991
GFI	>0.9	0.955
AGFI	>0.9	0.937

The path coefficients are shown in Table 6. Membership (MEM) (β = 0.269, *p*-value < 0.001), Influence (INF) (β = 0.237, *p*-value < 0.001) and Immersion (IMM) (β = 0.314, *p*-value < 0.001), are positively associated with affective commitment (AC). Therefore, hypothesis 1a, hypothesis 1b, and hypothesis 1c are verified. Affective commitment (AC) (β = 0.449, *p*-value < 0.001) is positively associated with user engagement (UE). Therefore, hypothesis 2 is verified.

**TABLE 6:** Regression coefficient.

Hypothesis	Unstd. coefficient	S.E.	*Z*-value	Std. coefficient	*P-value*
Hypothesis 1a: MEM->AC	0.247	0.058	4.232	0.269	***
Hypothesis 1b: INF->AC	0.206	0.055	3.750	0.237	***
Hypothesis 1c: IMM->AC	0.299	0.068	4.386	0.314	***
Hypothesis 2: AC->UE	0.339	0.050	6.768	0.449	***

***P-value < 0.001; MEM, membership; INF, influence; IMM, immersion; AC, affective commitment; UE, user engagement.

The results of the indirect effect analysis are shown in Table 7. In this study, structural equation modeling was used to analyze the indirect effect using Bootstrap estimation technique, and then the significant level of the indirect effect was further calculated. The indirect effect of membership (MEM) on user engagement (UE) is 0.084. At the 95% confidence level, “0” does not include the Bias-corrected 95% confidence interval range, the z-value > 1.96, and the *p*-value < 0.05. Therefore, there is an indirect effect exists. In the same analytical approach, the results of the study show that H3a, H3b, and H3c are significant.

**TABLE 7:** The analysis of indirect effect.

Indirect effect	Path coefficient	Bootstrap 1,000 times			
		Bias-corrected 95%		Percentile 95%	
		Lower bound	Upper bound	Lower bound	Upper bound
MEM→AC→UE	0.084	0.032	0.175	0.032	0.174
INF→AC→UE	0.070	0.026	0.122	0.025	0.119
IMM→AC→UE	0.101	0.046	0.167	0.041	0.158

*MEM, membership; INF, influence; IMM, immersion, AC, affective commitment; UE, user engagement.*

The moderating effects are reported in Table 8. In the present study, perceived support (PS) is the moderating variable. The results of structural equation modeling have been shown that the moderator effect of affective commitment (AC) × perceived support (PS) on user engagement (UE) is 0.083 (z = | 3.524| > 1.96, *p*-value < 0.001), implying the presence of a positive moderating effect of perceived support (PS) on the relationship between affective commitment (AC) and user engagement (UE). Specifically, the slope of affective commitment (AC) on user engagement (UE) increases positively by 0.083 units for each 1-unit increase in the moderating variable perceived support (PS). That is, perceived support (PS) has a positive moderating effect. Therefore, hypothesis 4 is verified.

**TABLE 8:** The analysis of moderating effect.

Dependent variable (DV)	Independent variable (IV)	Path coefficient (β)	S.E.	*Z*-value	*P-value*
UE	AC	0.345	0.052	6.605	***
	PS	0.054	0.040	1.362	ns
	AC × PC	0.083	0.023	3.524	***

UE, user engagement; AC, affective commitment; PS, perceived support. ***P-value < 0.001; ns, non-significant.

## Research and Discussion

### Findings and Discussion

First, the results of data analysis showed that sense of virtual community had a positive effect on affective commitment. The findings are consistent with the studies of Tiwana and Bush (2005), Hsu and Lin (2008), Zhou et al. (2012), Dai and Salam (2014), Hashim and Tan (2015), and Dennis et al. (2016). Zhao et al. (2012) showed that sense of virtual community has a significant effect on both knowledge acquisition and willingness to share knowledge, and the effect on the latter exceeds the effect on the former. Welboume et al. (2013) also found that due to the presence of members’ sense of attachment and responsibility, sense of virtual community may lead to sustained participation of community members. Especially when the presence of learning leaders in the community, teachers’ knowledge base, evaluation methods, the community’s resource base, platform features, learning facilities, and fast and slow internet speeds stimulate members’ membership, influence and immersion, it is more likely to stimulate consumers’ affective commitment to the educational virtual community. In addition, the harmonious relationships among community members, interactions in the community, emotional connections in the community, respect in the community, and sense of belonging in the community have a great impact on the affective tendencies of members in the educational virtual community. Therefore, the stronger the sense of virtual community created in the educational virtual community, the more practical benefits it provides to its members, and the more it motivates users to have affective commitment to the educational virtual community.

Second, the results of the data analysis indicate that affective commitment has a positive and positive effect on user engagement. The results of the study are consistent with the logical reasoning of Casaló et al. (2008); Brodie et al. (2013), Schulten and Schaefer (2015), and Jayasingh (2019). Emotion is a prerequisite for the experience of the existence of educational virtual community. Emotional response and user engagement are highly correlated, and user engagement is not a construct separate from emotional response, but actually a subjective psychological state of being involved, occupied, completely attracted, and fully absorbed by the educational virtual community, which can produce a specific attraction or repulsion for similar products. Members’ affective commitment is a psychological representation of their need for online learning, and the degree of need is related to the strength of integration. Values are members’ judgments about their learning needs and motivate them to choose what they think is important or valuable. In educational virtual communities, active choice is better than passive acceptance for members, and the duration of online learning, the effort of assignments or tasks, and the value of activities have a significant impact on the integration of members. Therefore, effective commitment plays an important role in the formation and development of user engagement, and there is a positive correlation between the two, and without a certain degree of effective commitment, it is impossible to successfully build an educational virtual community. Without a certain level of effective commitment, it is impossible to successfully build an educational virtual community.

Third, the results of data analysis indicate that affective commitment has mediating utility in the relationship between sense of virtual community and user engagement. The findings are consistent with the logical reasoning of Jang et al. (2008); Chang and Chuang (2011), Yang et al. (2014); Cheng and Guo (2015), and Shen et al. (2018). In educational virtual communities, member behavior is driven more by emotions than by interests. The social nature possessed by emotional responses is a necessary factor for the construction of virtual educational communities. In virtual educational communities, user engagement behavior represents the intention of members to socialize with others; affective commitment constructs and determines the degree of relationship and socialization with others and presents the response tendency of sense of virtual community. Specifically, in a virtual educational community, the sense of virtual community among members has a strong emotional orientation, and there is a strong connection between the sense of virtual community of members and the emotions they invest in learning. At the same time, users with emotional attachment have an ongoing desire to maintain relationships and bind themselves to the educational virtual community based on this willingness to become integrated into it. The higher the participation behavior of members in the virtual educational community, especially the participation behavior with positive emotions, the higher the perception of learning behavior and the higher the degree of user engagement. Therefore, the mediating effect of affective commitment is significant.

Fourth, the results of the data analysis indicate that perceived support has a moderating utility in the relationship between affective commitment and user engagement. The findings are inconsistent with the logical reasoning of Casaló et al. (2008); Brodie et al. (2013), Schulten and Schaefer (2015), and Jayasingh (2019) perceived support did not negatively moderate the relationship between affective commitment and user engagement. This may be due to the fact that in educational virtual communities, managers (e.g., teachers) strengthen user engagement through active participation, effective guidance, and moderate praise or encouragement of members. In other words, educational virtual communities reduce the negative effects caused by perceived support through corresponding. In other words, the educational virtual community reduces the negative effects of perceived support through appropriate organizational and managerial activities. Thus, perceived support, as an anxiety and concern of consumers in the educational virtual community, although inevitably hinder user engagement and the impact of effective commitment on consumer. However, the value created by the educational virtual community enables consumers to find like-minded friends and achieve optimized psychological needs in the educational virtual community, generating high levels of user engagement and offsetting the impact of perceived support in this process. The value created by educational virtual communities allows consumers to find like-minded friends and achieve optimized psychological needs in educational virtual communities, generating high levels of user engagement and offsetting the negative impact of perceived support in the process. In addition, the effect of the educational virtual community’s timely organization of offline gatherings of learners on the integration of members in the educational virtual community is extremely significant, which is the main reason for the insignificant moderating effect of perceived support.

### Theoretical Contributions

First, this study constructs a research model of the relationship between sense of virtual community, effective commitment, and user engagement. The findings of this study continue and corroborate the previous research on the behavior of educational virtual community members. As Casaló et al. (2008) points out, in the absence of real interaction, sense of virtual community is crucial to the formation and development of effective commitment. In educational virtual communities, effective commitment is a long-term mechanism for building relationships between members and the community, and the formation of such a mechanism cannot be achieved without the company’s community building. Sense of virtual community, as a subjective feeling of members toward the community, is the key to the formation and development of member commitment.

Second, user engagement, as a deeper and more meaningful manifestation of user-enterprise relationship orientation, is fundamentally different from the previous consumer psychology and behavior of merely being loyal to a brand and repeatedly buying branded products and services. Although scholars have begun to focus on the mechanisms of user engagement in virtual environments, their research has been limited to the interaction between firms and consumers. In fact, the formation of user engagement involves not only enterprises but also the perception of proposed authenticity given by the community to its members. This paper draws on the idea of social proximity theory to study the specific mechanism of sense of virtual community on the formation of user engagement, which provides a new idea for the study of the formation process of user engagement.

Third, this paper further enriches the study of affective commitment. Most of the previous studies on educational virtual community are based on member participation level, and the research models are scattered and lack theoretical foundation. The important concept of affective commitment originates from the field of organizational behavior. It has been pointed out that affective commitment is an enabler to motivate members’ participation (Cheng and Guo, 2015). As a powerful contractual force, affective commitment is the link between members and the educational virtual community. This study not only explores the impact of effective commitment on user engagement, but also further investigates the mediating role of effective commitment in the relationship between sense of virtual community and user engagement. This study has a certain reference value for enriching and developing academic theoretical research on educational virtual community at home and abroad, and also has a certain guiding role for related companies to improve the governance strategy of educational virtual community.

### Practical Implications

First, managers should focus on cultivating a good perception of educational virtual community among consumers. The sense of virtual community formed by consumers in educational virtual community has an impact on consumers’ attitudes and behaviors. In building, developing, and maintaining educational virtual communities, managers should pay attention to empowering consumers with good perceptions of educational virtual communities. Administrators should pay more attention to the management of virtual communities and try to create a good community atmosphere. When building educational virtual community, create a perfect information exchange mechanism for members, build a powerful network platform, and create conditions for communication among members. From the results of the study, managers should especially focus on fostering membership, and actively organize various activities for close communication among members so that they can develop a sense of membership to the community. In addition, certain feedback mechanisms should be designed to enhance the influence and immersion of community members. For example, for influence, this study can set up membership level privileges to strengthen members’ perception of influence; for immersion, this study can set up a check-in mechanism to increase the frequency of members’ visits to the community.

Second, managers should not ignore the expression of consumers’ attitudes in the educational virtual community. The results of this paper show that consumers’ affective commitment to educational virtual community connects consumers’ user engagement. Therefore, establishing a reasonable attitude expression mechanism and strengthening consumers’ affective attitude expression influence the development and prosperity of educational virtual community’s development and prosperity. Administrators should focus on maintaining the emotional connection between members and the community, so that members see it as their duty to create content. When managing an educational virtual community, it is important to create a “family” culture. For example, this study should set up a self-governance mechanism for the community members so that they can manage themselves. This study should also support virtual and physical interactions between members with high level online and offline technology development teams to provide a good communication space for members. In addition, strengthening the offline interactions of the educational virtual community should not be overlooked. For example, you can invite some members to visit companies and send greetings on holidays to strengthen the emotional connection with members.

Third, managers should be aware of the influence of some weighting factors on the behavior of educational virtual community consumers and adopt different strategies for different user groups. For members with high level of perceived support, they should highlight the mechanism of fostering their effective commitment, not only encouraging members and other community members to form a high level of effective commitment through continuous interaction, but also focusing on cultivating the effective commitment of community members In order to establish the unique values and cultural atmosphere of the educational virtual community, this study should establish clear boundaries between the educational virtual community and other similar communities, consolidate and maintain the effective commitment of users, and promote user engagement with the educational virtual community. In addition, managers should pay attention to stimulate members to voluntarily carry out activities that focus on the brand community, as well as to assist in the operation of the community platform and actively disseminate brand products and other specific behaviors, such as word-of-mouth communication, recommendations, feedback, and evaluation, etc., reflecting the intimate relationship between members’ self-concept and the brand community, thereby strengthening the competitive advantage of the educational virtual community. This strengthens the competitive advantage of the educational virtual community. For example, managers can also convert the points and experiences accumulated by consumers in the community into vouchers for purchasing products, and further expand the scope of application of the educational virtual community, such as helping its members to solve problems outside the community, and continuously increase the benefits that members can obtain in the educational virtual community. The benefits that members can obtain in the educational virtual community will be continuously increased.

### Research Limitations and Future Research Directions

On the one hand, in the sample of this study, the representativeness of the sample in terms of education and marriage needs to be further enhanced. Although this study strictly follows the analysis in strict accordance with the requirements of the questionnaire survey as well as the empirical research, there is a bias in the sample source for higher education and married, which may reduce the generalizability of the study findings. Considering the possible differences in perceptions of the connotations of sense of virtual community among different marital and educational groups, it is possible that there are different predictions of the effects of sense of virtual community on behavior based on different users. Therefore, future studies can expand the scope of data collection, consider the representation of gender and education in the sample, and conduct structural equation modeling studies with larger samples to enhance the generalizability of the findings.

On the other hand, in the study of the impact of sense of virtual community on user engagement, this study explored the mediating role of affective commitment and the moderating role of perceived support. Although affective commitment and perceived support play an important research value in the relationship between sense of virtual community and user engagement, the mediating and moderating variables that affect sense of virtual community on user. The mediating and moderating variables that influence sense of virtual community on user engagement are not limited to these variables. Therefore, more empirical studies are needed to further explore the mediating mechanisms and boundary conditions of user engagement in the context of educational virtual community use. Given the importance of the research topic, future studies could attempt to extend the theoretical model proposed in this study by incorporating other potential moderating and mediating variables.

## Data Availability Statement

The original contributions presented in this study are included in the article/supplementary material, further inquiries can be directed to the corresponding author.

## Ethics Statement

Ethical review and approval was not required for the study on human participants in accordance with the local legislation and institutional requirements. Written informed consent was obtained from all participants for their participation in this study.

## Author Contributions

HZ: conceptualization and writing original draft. QS: formal analysis and investigation. Both authors contributed to the writing – review and editing and read and agreed to the published version of the manuscript.

## Conflict of Interest

The authors declare that the research was conducted in the absence of any commercial or financial relationships that could be construed as a potential conflict of interest.

## Publisher’s Note

All claims expressed in this article are solely those of the authors and do not necessarily represent those of their affiliated organizations, or those of the publisher, the editors and the reviewers. Any product that may be evaluated in this article, or claim that may be made by its manufacturer, is not guaranteed or endorsed by the publisher.
